# Prevalence of carotid artery calcifications among 2,500 digital panoramic radiographs of an adult Brazilian population

**DOI:** 10.4317/medoral.22350

**Published:** 2018-04-24

**Authors:** Juliana-Mara-Oliveira Santos, Guilherme-Costa Soares, Ana-Paula-Negreiros-Nunes Alves, Lúcio-Mitsuo Kurita, Paulo-Goberlânio-de Barros Silva, Fábio-Wildson-Gurgel Costa

**Affiliations:** 1 Departament of Dentistry Clinical, Federal University of Ceará, Fortaleza, Brazil

## Abstract

**Background:**

The aim of the present study was to analyze the epidemiological data of digital panoramic radiographs revealing suggestive images of carotid artery calcifications (CAC) from a Northeast Brazilian population.

**Material and Methods:**

A cross-sectional retrospective study was conducted with 2,500 digital panoramic radiographs obtained from a single imaging reference center in Northeast Brazil. Images from individuals of both sexes and older than 18 years were included and those that did not cover the region of cervical vertebrae or presented low radiographic quality were excluded. Data were analyzed regarding prevalence, location (bilateral, right or left), sex, and age using the Chi-square test at the significance level of 5%.

**Results:**

An amount of 96 (4%) patients presented suggestive images of CAC. The female sex (*p*=0.003) and individuals aged up to 70 years (*p*=0.002) were statically significant. 40.4% were found bilaterally, 37.6% on the right side (*p*<0.001) and 22% on the left side.

**Conclusions:**

In conclusion, this study showed a low prevalence of suggestive images of CAC in digital panoramic radiographs from a Northeast Brazilian population. It was observed a higher prevalence of CAC associated with female sex, older patients, and right side location.

** Key words:**Atherosclerosis, carotid artery, digital panoramic radiograph, prevalence.

## Introduction

Coronary artery disease is considered a major cause of worldwide morbidity and mortality (1), being a multifactorial disorder in which multiple genetic variants are combined with several environmental risk factors and deleterious lifestyles (2). It is characterized by the long-term formation of atheromatous plaques within the arterial walls, culminating in atherothrombotic obstructive lesions leading to local tissue damage, such as ischemic stroke, transient ischemic attack, or amaurosis fugax (3). These aspects make the presence of atherosclerotic plaques a relevant health problem since heart attack is one of the main causes of death in the world, as well as it is closely related to severe impairments for the patients affected by this disease (4).

Carotid artery calcifications (CAC) can be visualized on panoramic radiographs, imaging exams that are routinely used in the evaluation of patients with dental issues (5). The presence of CAC in these exams is an indicator of the risk of developing possible cardiovascular events, which justifies the clinical interest of the dentist in being able to identify these alterations early (6). These alterations appear as single or multiple, non-continuous nodular radiopaque images, located on the intervertebral junction C3-C4, about 1 to 2.5 cm inferior to posterior to the angle of the mandible, or as vertical radiopaque lines representing thin vascular wall calcifications (7,8).

The differential diagnosis of atherosclerotic plaques includes anatomical structures of the neck region such as hyoid bone, trichoid cartilage, the superior horn of the calcified and epiglottic thyroid cartilage, and lesions such as sialoliths, elongation, and calcifications of the styloid process, calcifications in the stylomandibular and stylohyoid ligaments, phleboliths, among others (5,6,8).

Epidemiological studies have shown that the prevalence of CAC found in panoramic radiographs, performed for dental reasons, is around 2% to 5% (9). However, a limited number of studies regarding CAC prevalence on digital panoramic radiographs from South America have been published to date. Thus, the present study aimed to evaluate the epidemiological data of digital panoramic radiographs showing suggestive images of CAC from a significant Brazilian population.

## Material and Methods

-Study design

A retrospective cross-sectional study was performed with a sample of 2500 digital panoramic radiographs obtained between December 2011 to December 2014 from a private dental imaging clinic, which is a reference service in the state of Ceará, Brazil. The present study was approved by the Research Ethics Committee under protocol number 285/11. It was used the Cranex D (Soredex, Tuusula, Finland) digital panoramic and cephalometric imaging system. The images were acquired with Frankfurt’s horizontal plane parallel to the ground and they were generated with a resolution of 300 dpi and setting parameters adjusted according to the patient’s size.

**Figure 1:**
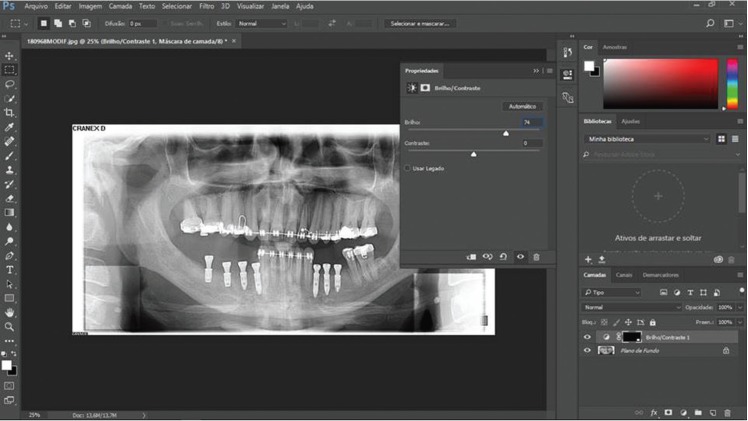
Adobe Photoshop® window showing the use of a filter with saturation and balance standardized in order to analysis a bilateral CAC on panoramic radiograph.

Images of individuals under the age of 18 years, that did not cover the region of cervical vertebrae, or that presented low radiographic quality, were excluded from this sample (n=111). Two previously calibrated examiners (kappa coefficient = 0.856) analyzed the radiographs in order to detect images suggestive of CAC. The images were analyzed using Adobe Photoshop® CC 2015 software (Adobe Systems Incorporated, California, USA) for saturation and contrast in order to standardize images during data evaluation (Fig. 1). CAC were considered present when it was observed heterogeneous radiopacities located into the intervertebral space between C3 and C4 (6). Before the radiographic analysis, two examiners were calibrated in order to establish uniform criteria for imaging evaluation. Cohen´s Kappa statistic was applied and it was obtained an inter-rater agreement value higher than 0.80. Doubts about the presence of CAC were interpreted by two oral and maxillofacial radiologists. The two radiologists analyzed 118 doubts of the panoramic radiographs being the main differential diagnosis the cricoid cartilage. The studied variables were sex, age, and location (bilateral, right unilateral or left unilateral).

-Statistical analysis

The data were tabulated in Microsoft Excel 2010 software regarding location (bilateral, right unilateral or left unilateral), gender and age in order to obtain data on the prevalence of calcifications and the results were submitted to statistical treatment by the Chi-square test at the significance level of 5%. Statistical Package for the Social Sciences (SPSS) software 15.0 version for Windows (SPSS Inc.®, Chicago, Illinois, USA) was used for all analysis.

## Results

In this study, CAC prevalence was 4% (n=96) considering a sample with an estimated power of 99.6%.  Table 1 showed a statically significant difference (*p*=0.003) between females (n=68; 70.8%) and males (n=28; 29.2%).

**Table 1:**
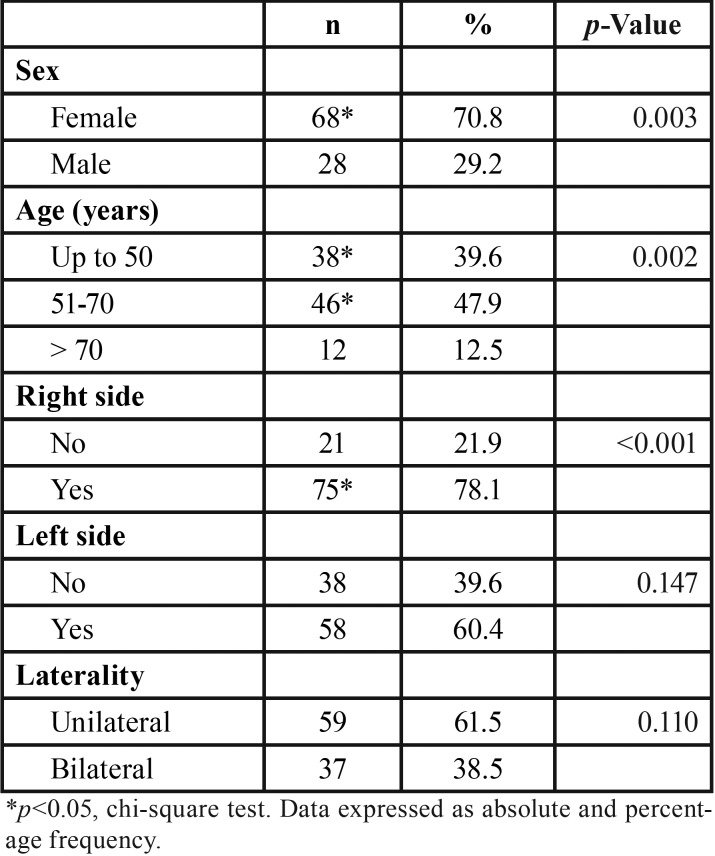
Characterization of the sample according to sex, age and location.

Radiographs suggesting CAC were observed in individuals with age ranging from 25 to 89 years (mean age of 54±13 years). There was no statically significant difference (*p*=0.968) between male (54±15 years) and female (54±12 years) mean age. In addition, sex and age did not show a statically significant association with the right, left or both sides (*p*>0.05). The most prevalent and statically significant (*p*=0.002) age group was that one between 50 and 70 years (n=46; 47.9%), followed by individuals aged up to 50 years (n=38; 39.6%).

There was a statically significant amount of patients presenting suggestive images of CACs on the right side (*p*<0.001). However, there was no difference regarding the occurrence even in the left side (*p*=0.147) or in a bilateral presentation (*p*=0.110). According to  Table 2, there was no statically significant association of sex with age groups (*p*=0.443), right side (*p*=0.635) or left side (*p*=0.379). Bilateral CAC did not show association with sex (*p*=0.198;  Table 1) or age (*p*=0.598;  Table 3).

**Table 2:**
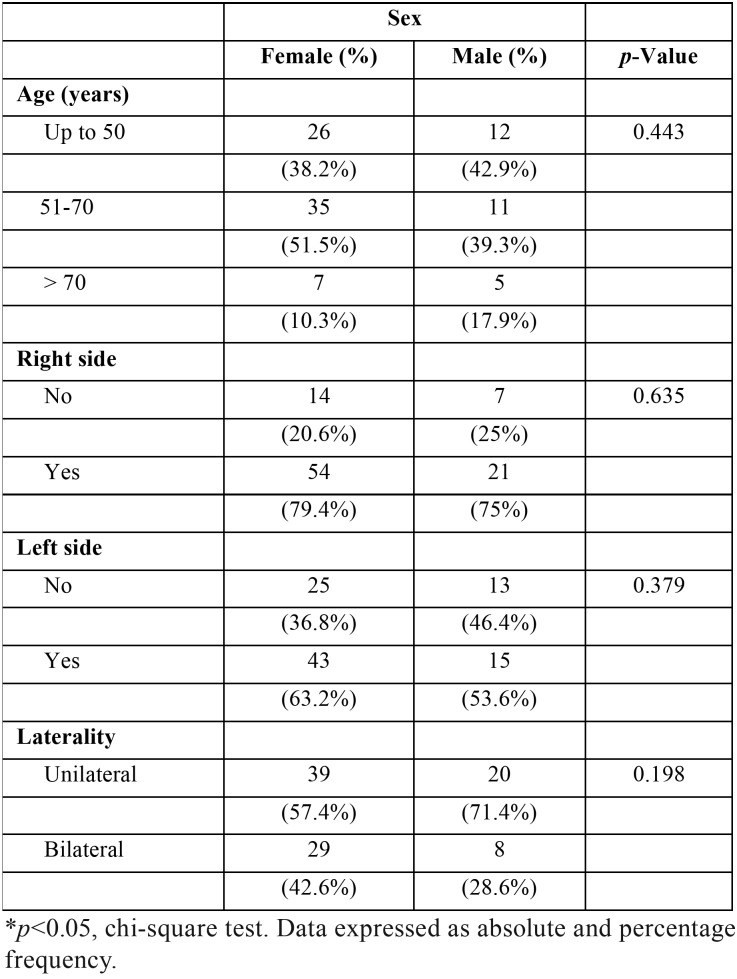
Characterization of sex according to age, side and laterality.

**Table 3:**
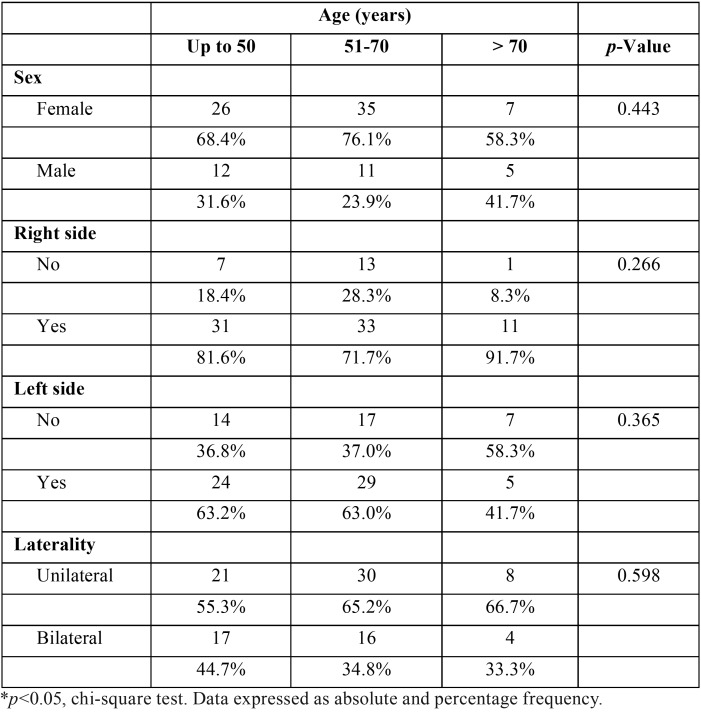
Characterization of the sample according to age versus sex, side and laterality.

## Discussion

Atherosclerotic plaques in carotid arteries have been extensively studied in the field of Medicine and Dentistry. Indeed, atherosclerotic plaques have been considered important predictors of cardiovascular disorders and, therefore, their identification could be useful as a tool for medical stratification and health education strategies according to Gepner *et al.* (10). In a systematic review with a meta-analysis published in 2017 by Gorgui *et al.* (3), the prevalence of atheromatous plaques differed according to geographic regions: South Africa (38.1%), United States (17.2-43%), Japan (29.8%), Europe (5.4%), Spain (60%), and China (44.4-51%).

In dentistry literature, Friedlander and Lander (1981) (7) were the first researchers to report CAC visualized on panoramic radiographs, emphasizing the importance of this exam since it is a routine radiograph required in dental practice and, in many cases, the identification of CAC is an accidental finding (5). These aspects motivated the conduction of the present research, also reinforced by the retrospective collection of a substantial number of digital panoramic radiographs from a single reference center for maxillomandibular imaging exams in Northeast Brazil.

Almog *et al.* reported that the prevalence of CAC observed by panoramic radiographs has been usually described up to 5% with higher occurrence in patients with clinical conditions associated with atherosclerosis (9). The present study showed a percentage of 4% in an adult Northeast Brazilian population and since it had a non-intentional sample recruitment, clinical data were not obtained, which may explain the different prevalence in comparison with other investigations. Regarding epidemiological data on the worldwide prevalence of CAC in panoramic radiographs, there have been reported similar results to the present study in Germany, Saudi Arabia, and Japan (11-13). In Brazil, the largest study that evaluated 8,338 panoramic radiographs was performed in the South region and it showed a CAC prevalence of 6.9% (14). Thus, the present study is the second one in Brazil that presented a significant sample size and it is the largest one from the Brazilian Northeast region to date.

In the present cross-sectional study, the age group between 50 and 70 years showed a statistical significance prevalence. A similar result was observed by Lee *et al.* (13) since the authors found a higher prevalence of images suggestive of atheromatous plaques in patients aged 40 to 70 years. These results reinforce the age as an important risk factor for CAC. In younger patients, only a small number of cases of carotid artery calcifications has been found. In spite of this finding, atheromatous plaques should be suspected at any age, justifying the evaluation of panoramic radiographs in younger patients (15).

This study showed a statistically significant prevalence of CAC in women, which has been commonly found in the literature. Friedlander and Altman (1981) (7) stated that this association may occur due to a decline in the estrogen level after menopause. Since it is a hormone that acts on lipoproteins metabolism by preventing the formation of atheromatous plaques, its reduced level may precipitate the formation of atheromas. Friedlander *et al.* (16) evaluated the panoramic radiographs of women with a history of amenorrhea greater than 12 months and images suggestive of CAC appeared in 31% of the radiographs. Patil *et al.* (17) reported a 22.9% prevalence of CAC in women aged over 50 years among a sample of 1,214 panoramic radiographs. Regarding occurrence of CAC in men, it has been considered that the smoking habit plays an important risk factor for its development, which differs in relation to women as described by Cohen *et al.* (18).

The right side showed a statistically significant prevalence in the present study. Similar findings were found by Ohba *et al.* (19), which revealed a prevalence of 74% of CAC on the right side. This result is reasonable according to aforementioned authors, which cited that the level of bifurcation of the right carotid artery is most often located between C3 and C4, whereas the left carotid artery shows a bifurcation mostly observed between C4 and C5.

In the present investigation, the differential diagnosis included radiopaque structures resembling atheromatous plaques on digital panoramic radiographs. CAC were considered in the presence of one or more non-continuous adjacent radiopaque nodular images or vertical lines at the intervertebral space between C3 and C4, and located posteriorly and lower than the mandibular angle (20,21). In addition, the present study included as potential differential diagnosis other conditions, including sialoliths, tonsilloliths, as well as stylohyoid ligament and triticeous cartilage calcifications, and the hyoid bone. Sialoliths are usually located in the submandibular gland or in its duct and have a radiographic pattern as a single or multiple calcifications. On panoramic radiographs, they can be seen superimposed on or below the body or ramus of the mandible, which is a most anterior location than observed in CAC. Tonsilloliths are small calcifications developed into tonsillar crypts and are described as radiopaque images superimposed on the mandibular branch on panoramic radiographs. The styloid process has been radiographically described as a cylindrical and radiopaque image projecting forward and down between the mandible ramus and the mastoid process. The hyoid bone is presented as a bilateral and horizontal well-defined radiopaque image located below the mandible. Calcified triticeous cartilage presents as an ovoid radiopaque image, about 2 to 4 mm wide and 7 to 9 mm long, normally found within the air space adjacent to the pharynx (21).

Regarding the study design adopted in this research, it was not possible to use the Doppler ultrasonography in order to confirm the diagnosis of CAC. However, in the field of Oral and Maxillofacial Radiology, we believe that is important to add literature concerning epidemiological data from different geographic locations.

In summary, the present study showed a low prevalence of suggestive images of CACs on digital panoramic radiographs from an adult South American subpopulation. It was observed a higher prevalence in women and older patients, and the right side was the main location for CAC.

## References

[1] Sulo E, Nygård O, Vollset SE, Igland J, Ebbing M, Østbye T (2017). Time Trends and Educational Inequalities in Out-of-Hospital Coronary Deaths in Norway 1995-2009: A Cardiovascular Disease in Norway (CVDNOR) Project. J Am Heart Assoc.

[2] Fawzy MS, Toraih EA, Aly NM, Fakhr-Eldeen A, Badran DI, Hussein MH (2017). Atherosclerotic and thrombotic genetic and environmental determinants in Egyptian coronary artery disease patients: a pilot study. BMC Cardiovasc Disord.

[3] Gorgui J, Gasbarrino K, Georgakis MK, Karalexi MA, Nauche B, Petridou ET (2017). Circulating adiponectin levels in relation to carotid atherosclerotic plaque presence, ischemic stroke risk, and mortality: A systematic review and meta-analyses. Metabolism.

[4] Olindo S, Saint-Vil M, Jeannin S, Signate A, Edimonana-Kaptue M, Cabre P (2016). One-year disability, death and recurrence after first-ever stroke in a Black Afro-Caribbean population. Int J Stroke.

[5] Friedlander AH, Garret NR, Norman DC (2002). The prevalence of calcified carotid artery atheromas on the panoramic radiographs of patients with type 2 diabetes mellitus. J Am Dent Assoc.

[6] Friedlander AH, Gratt BM (1994). Panoramic dental radiography as an aid in detecting patients at risk for stroke. J Oral Maxillofac Surg.

[7] Friedlander AH, Lande A (1981). Panoramic x-ray identification of carotid arterial plaques. J Am Dent Assoc.

[8] Friedlander AH, Manesh F, Wasterlain C (1994). Prevalence of detectable carotid artery calcification on panoramic radiographs of recent stroke victims. J Am Dent Assoc.

[9] Almog DM, Horev T, Illig KA, Green RM, Carter LC (2002). Correlating carotid artery stenosis detected by panoramic radiography with clinically relevant carotid artery stenosis determined by duplex ultrasound. Oral Surg Oral Med Oral Pathol Oral Radiol Endod.

[10] Gepner AD, Young R, Delaney JA, Budoff MJ, Polak JF, Blaha MJ (2017). Comparison of Carotid Plaque Score and Coronary Artery Calcium Score for Predicting Cardiovascular Disease Events: The Multi-Ethnic Study of Atherosclerosis. J Am Heart Assoc.

[11] Tiller R, Bengel W, Rinke S, Ziebolz D (2011). Association between carotid area calcifications and periodontal risk: a cross sectional study of panoramic radiographic findings. BMC Cardiovasc Disord.

[12] Alzoman HA, Ra'ed I, Al-Lahem Z (2012). H, Al-Sakaker AN, Al-Fawaz Y F. Prevalence of carotid calcification detected on panoramic radiographs in a Saudi population from a training institute in Central Saudi Arabia. Saudi Med J.

[13] Lee JS, Kim OS, Chung HJ, Kim YJ, Kweon SS, Lee YH (2013). The prevalence and correlation of carotid artery calcification on panoramic radiographs and peripheral arterial disease in a population from the Republic of Korea: the Dong-gu study. Dentomaxillofac Radiol.

[14] Gonçalves JR, Yamada JL, Berrocal C, Westphalen FH, Franco A, Fernandes Â (2016). Prevalence of Pathologic Findings in Panoramic Radiographs: Calcified Carotid Artery Atheroma. Acta Stomatol Croat.

[15] Bayer S, Helfgen EH, Bös C, Kraus D, Enkling N, Mues S (2011). Prevalence of findings compatible with carotid artery calcifications on dental panoramic radiographs. Clin Oral Invest.

[16] Friedlander AH, Altman L (2001). Carotid artery atheromas in postmenopausal women: their prevalence on panoramic radiographs and their relationship to atherogenic risk factors. J Am Dent Assoc.

[17] Patil SR (2015). Prevalence of carotid artery calcification in postmenopausal women and its correlation with atherogenic risk factors. J Nat Sci Biol Med.

[18] Cohen SN, Friedlander AH, Jolly DA, Date L (2002). Carotid calcification on panoramic radiographs: an important marker for vascular risk. Oral Surg Oral Med Oral Pathol Oral Radiol Endod.

[19] Ohba T, Takata Y, Ansai T, Morimoto Y, Tanaka T, Kito S (2003). Evaluation of calcified carotid artery atheromas detected by panoramic radiograph among 80-year-olds. Oral Surg Oral Med Oral Pathol Oral Radiol Endod.

[20] Lewis DA, Brooks SL (1999). Carotid artery calcifiction in a general dental population: a retrospective study of panoramic radiographs. Gen Dent.

[21] Friedlander AH, Friedlander IK (1998). Identification of stroke prone patients by panoramic radiography. Aust Dent J.

